# Hospital Expenditure.—The Commissariat

**Published:** 1896-09-12

**Authors:** 


### The Institutional Workshop.

### HOSPITAL EXPENDITURE.—THE COMMISSARIAT.

### XXVI.—THE BUTTERMAN'S BILL.

Under this heading in the uniform system of accounts are classed cheese, butter (salt and fresh), lard, bacon, and ham. Even in those hospitals, however, where the uniform system is observed, there is a wide difference in practice as to what is included in this item. Among London hospitals eggs are included at the Great Northern Central, the Metropolitan and St. Thomas's. Butter, cheese, and eggs find no place in the accounts at Addenbrooke's or at the Sussex County, and are presumably included with poultry or grocery, and it is very common to find eggs included with butter, and ham and bacon reckoned as meat. In some provincial hospitals, however, the satisfactory plan is adopted of dissecting the butterman's bill, and stating the amount expended on butter, cheese, bacon, and ham separately. It may be as well to observe on this point that, although the uniform system of accounts has set up a standard minimum of information to be offered to the public, it by no means places limits on the items to be mentioned, and the plan of detailing the cost of all the main articles of diet separately has many advantages, while it entails no additional labour on a well-managed secretarial department.

Only one of the items in the butterman's bill enters into the patients' bill of fare, viz., butter, and even this is not always provided for him. In institutions where butter is provided the patient usually receives 1 oz. a day, amounting for each occupied bed to 23 lbs. a year. The price paid for butter varies less than for many other articles, 1s. or 1s. 1d. being the more ordinary London price for patients, and 1s. 2d. the average in other places. From 23s. to 25s. may then be considered the average cost of each occupied bed for this article of diet. At Guy's the yearly cost per patient works out at £1 2s. All patients are not, it is true, on bread and butter diet, but even those on milk commonly receive a small amount, so that it is impossible to reckon on much reduction for the percentage on low diet. A certain amount of butter, not less than a pennyworth a week each, must be allowed for cooking, and this will about balance any saving on non-butter eating patients.

As regards the household, it is difficult to establish a fair mean of expenditure. To determine how much should be allowed for different sections of the household is always a harassing point for a new housekeeper, and all preconceived theories are apt to scatter to the winds in the face of a few weeks' experience. The following computation actually coincides in its total with the year's cost per head of the butterman's bill at Guy's for nurses and household:—

| | | | | | |
|---|---|---|---|---|---|
| Butter, ½ lb. per week, at 1s. 1d. ... | yearly | £1 | 10 | 0 |
| Bacon, ½ lb. „ at 0s. 9d. ... | „ | 1 | 0 | 0 |
| Cheese, ½ lb. „ at 0s. 6d. ... | „ | 0 | 13 | 0 |
| Cooking butter or lard, 3d. per week... | „ | 0 | 13 | 0 |
| Total for year ... ... ... | | £3 | 16 | 0 |

This should prove a liberal allowance, but it may be modified indefinitely by the extent to which tinned meats, fish, eggs, and jam are used. For instance, if bacon were the only breakfast dish for the week, ½ lb. per head is not nearly enough; but in most institutions some variety or choice is provided. Again, bread and cheese lunches or suppers will, of course, send up the cheese bill to a figure unknown in institutions where porridge, sardines, potted meat, or puddings take its place.

In the following London hospitals where butter is provided for the patients, deduction has been made for the cost of this at the rate of 25s. a year per occupied bed, and the remainder of the butterman's bill has been divided among the household:—

| | Cost of Patients' Butter at 25s. a year per bed. | Surplus for Butter, Bacon, Cheese, divided among Household. |
|---|---|---|
| Seamen's ... ... | £267 ... | ... £1 10 0 |
| Charing Cross... ... | 187 ... | ... 2 9 0 |
| St. George's ... ... | 403 ... | ... 3 7 0 |
| St. Mary's ... ... | 313 ... | ... 5 18 0 |

In the following hospitals butter is not provided for the patients. An average of 4s. a year has been deducted for each occupied bed to cover cost of butter for cooking, and the remainder of the bill for butter, cheese, and bacon divided among the household:—

| | Cost of Patients at 4s. a year. | Yearly average for Household. |
|---|---|---|
| University ... ... | £37 ... | ... £2 10 0 |
| Middlesex ... ... | 43 ... | ... 3 0 0 |
| London Hospital ... | 131 ... | ... 4 10 0 |
| *Royal Free ... ... | 34 ... | ... 4 8 0 |

* Including butter for 10 per cent. of patients.

At the London Hospital Nursing Home the average is £4 yearly. Turning now to provincial institutions, it becomes possible to observe in some instances the exact quantities per head of these articles consumed in the year, and in others the actual cost per head, neither of which is it possible to do more than guess at in the case of the London institutions above named.

In the following hospitals butter is provided for the patients, and the amount used has been, therefore, divided equally among all the inmates:—

| | Yearly quantity of Butter used by each Inmate. |
|---|---|
| Halifax ... ... ... ... | 22 lbs. |
| Norfolk and Norwich ... ... | 22 lbs. |
| Belfast Royal ... ... ... | 22 lbs. |
| Leeds General Infirmary ... ... | 25 lbs. |
| Sussex County ... ... ... | 26 lbs. |
| Oxford, Radcliffe ... ... ... | 29 lbs. |

At the Bristol General Infirmary and Birmingham Queen's, where butter is not provided for the patients, the amount used, divided among the household, is respectively 32 lbs. and 37 lbs. per head (making no deduction for cooking).

The following table gives the yearly cost of butter, bacon, and cheese per head in some institutions where these items are stated separately in the accounts. The cost of butter is divided among the inmates, while that of cheese and bacon is shared only among members of the household:—

| | Average Yearly Cost of Butter per Inmate. £ s. d. | Average Yearly Cost of Bacon and Ham—Household. £ s. d. | Average Yearly Cost of Cheese—Household. s. d. |
|---|---|---|---|
| Oxford Radcliffe ... | 1 12 0 ... | 0 17 0 ... | 11 0 |
| Norfolk and Norwich | 1 6 0 ... | 1 12 0 ... | 2 0 |
| Halifax ... ... | 1 4 0 ... | 1 12 0 ... | 5 0 |
| Birmingham Queen's | — ... | 1 0 0 ... | 4 0 |
| Bristol General ... | — ... | — ... | 12 0 |
| York County ... | 1 5 0 ... | 1 9 0 ... | 5 0 |
| Edinburgh Roy. In. | 1 2 0 ... | 1 11 0 ... | 7 0 |
| Belfast Royal ... | 1 6 0 ... | 1 1 0 ... | 0 6 |

The price of butter provided for the patient averages in London 121s. 4d. per cwt., or 1s. 1d. per lb. At St. Bartholomew's and Middlesex separate quotations are given for summer and winter, but it is more usual, though undoubtedly less satisfactory, to contract for an all-round supply at one price. As little as 96s. 7d. or 10¼d. per lb., is given at one hospital and 98s. per cwt. at another. The cost of butter in the country towns nowhere falls below 1s. 1d., and in some places rises to 1s. 3d. or 1s. 4d. The average is 1s. 2d. In Scotland the price is again rather lower, from 1s. per lb. to 1s. 1½d. The prices paid for bacon range in London from 7½d. to 10d. per lb., and in the country from 6d. to 9d. In Scotland the prices vary from 6½d. to 10d.

It is a matter for much regret that in so many hospitals a decided distinction is drawn in the quality of food provided for different classes of inmates. In two or three London hospitals three qualities of butter are provided, presumably for doctors, nurses, and patients' consumption. That the doctors should be provided with victuals of a more delicate quality than the nurses is an evil, but it is an evil admitting of the explanation that these gentlemen work on the unsatisfactory basis of receiving board in lieu of salary and are naturally a trifle "exigeants." But that the patients should be placed on a lower level than any of the rest of the household in respect of the quality of their food is not to be defended on any principle of economy or expediency. In one or two London hospitals the price quoted for the patients' butter is altogether unjustifiable; to offer to the recipients of the most munificent charities in the world food so unpalatable that hard-working men and women turn away from it with disgust, is an instance of ill-judged parsimony which no committee should sanction.

There are happily some London and many provincial institutions where care is taken to procure good butter at one price for all the inmates. Do we not all know the nice power of discrimination which the poor possess in the matter of tea and butter, and the painful eagerness with which the hour for meals is awaited in the long uneventful hospital day? Is it too much to plead that the bit of really good butter which is so highly valued should lend a zest to what is at best a somewhat monotonous repast? One more calculation. A difference of 2d. per lb. lies between the good and the bad; reckoning then for each bed an average of 20 lbs. consumed in the year (exclusive of the allowance for cooking), the extra cost of the better quality would be less than £17 for each 100 beds. And how easily far more than this might be saved in other directions affecting no one's comfort, readers of "The Commissariat" may judge.

